# Translational Significance for Tumor Metastasis of Tumor-Associated Macrophages and Epithelial–Mesenchymal Transition

**DOI:** 10.3389/fimmu.2017.01106

**Published:** 2017-09-13

**Authors:** Wenzhe Song, Roberta Mazzieri, Tao Yang, Glenda C. Gobe

**Affiliations:** ^1^Faculty of Medicine, Translational Research Institute, The University of Queensland Diamantina Institute, Brisbane, QLD, Australia; ^2^Department of General Surgery, Affiliated Hospital of Xuzhou Medical University, Xuzhou, China; ^3^Discipline of Pathology, The Western Sydney University, Sydney, NSW, Australia; ^4^SydPath, St Vincent’s Hospital, Sydney, NSW, Australia

**Keywords:** tumor microenvironment, tumor-associated macrophages, epithelial–mesenchymal transition, metastasis, EMT

## Abstract

The tumor microenvironment determines development and progression of many cancers. Epithelial–mesenchymal transition (EMT) is fundamental to tumor progression and metastasis not only by increasing invasiveness but also by increasing resistance to cell death, senescence, and various cancer therapies; determining inflammation and immune surveillance; and conferring stem cell properties. It does this by enabling polarized epithelial cells to transform into cells with a mesenchymal, and therefore motile, phenotype. Tumor-associated macrophages (TAMs) are key cells of the tumor microenvironment that orchestrate the connection between inflammation and cancer. Activation of EMT often requires crosstalk between cancer cells and components of the local tumor microenvironment, including TAMs. In this review, clinical and experimental evidence is presented for control of TAMs in promoting cancer cell invasion and migration and their interaction with the EMT process in the metastatic cascade. The translational significance of these findings is that the signaling pathways that interconnect TAMs and EMT-modified cancer cells may represent promising therapeutic targets for the treatment of tumor metastasis.

## Introduction

Tumor metastasis is responsible for 90% of cancer-related mortality but is still one of the most poorly understood components of cancer pathogenesis (1). The majority of solid tumors are carcinomas, cancers that originate in the epithelial cell population. Successful invasion-metastasis cascades require several steps that include epithelial–mesenchymal transition (EMT) of cancer cells, invasion through the extracellular matrix (ECM) and stromal cell layers, intravasation into the vasculature lumina, transport through the circulatory system, extravasation into parenchyma of distant tissues and organs, seeding at the premetastatic niche, and finally survival and growth at the metastatic site (1, 2). Chaffer, Weinberg and colleagues have described the complicated and multistep metastatic process in two stages. In the first stage, cancer cells translocate physically from the primary tumor to the site of dissemination; and in the second stage, colonization occurs at the secondary site (3–5). As simple as these processes may sound, the clinical impact of these changes is immense. Many of the events involved in these stages are the result of reciprocal and evolving crosstalk between the tumor microenvironment and carcinoma cells. The EMT program plays an important role in tumor metastasis by disassembling adherens and tight junctions, transforming polarized epithelial cancer cells into a mesenchymal cell phenotype, and facilitating the detachment of mesenchymal cells from initial sites to allow passage through dismantled basement membranes (BMs) (Chaffer and Weinberg’s stage 1) (4). Once they have reached the distant organs (Chaffer and Weinberg’s stage 2), these mesenchymal cells may return to an epithelial phenotype through a mesenchymal–epithelial transition (MET) and thereby regain the ability of cancer cell proliferation and differentiation in metastatic sites (4).

Activation of the EMT program for metastasis typically requires crosstalk between cancer cells and the local microenvironment. Different populations of chronic inflammatory cells, some of which mature to be tumor-associated macrophages (TAMs), influence the local tumor microenvironment. These inflammatory cells play an important role in tumor progression by promoting tumor cell proliferation, matrix remodeling, angiogenesis, suppressed adaptive immunity, and EMT (2, 6). Classically, they do this by producing various cytokines such as the interleukins (ILs), interferon (IFN), and other tumor-promoting factors (7, 8). Research that is more recent has now demonstrated that EMT-mediated premetastatic tumor cells are also involved in the recruitment, activation, and differentiation of TAMs (5). The fundamental concept of EMT stimulating cancer development and progression is well accepted, but the links between EMT and TAMs in these processes present a gap in research. For example, although the tumor inflammatory cell populations appear to promote growth and progression of tumors, the exact interactive pathways are not well defined.

By defining the roles of EMT and TAMs in cancer development, therapeutic strategies that target these processes may be developed to benefit cancer patients (9). The purpose of this review is to provide an overview of experimental and clinical evidence that demonstrates the crosstalk between TAMs and cancer cells undergoing EMT during metastasis and develop an understanding of the translational significance of this information, for development of new diagnostic and therapeutic strategies targeting TAMs and/or EMT.

## EMT in Tumor Metastasis

Epithelial–mesenchymal transition refers to a series of biologic processes that allow polarized epithelial cells, which normally adhere to the BM, to undergo multiple biochemical and molecular changes to transform into mesenchymal-like cells (5, 10, 11). The hallmarks of EMT include loss of cell-to-cell adhesion; loss of apical–basal polarity; and acquisition of increased migratory and invasive properties. There are three different classifications of EMT: type 1 EMT is involved in embryo implantation, embryogenesis, and organ development; type 2 EMT is a process involved in wound healing, tissue regeneration, and organ fibrosis, initiated by the injury; and type 3 EMT is associated with cancer progression and metastasis (12). In a comprehensive review of EMT in cancer development, Micalizzi et al. (13) suggest that cancer cell motility may also use a process of non-EMT epithelial cell plasticity, termed collective migration. The similarities between EMT and collective migration suggest that the epithelial and mesenchymal cell phenotype transition may not always involve two absolute and independent cell states. Instead, a continuous spectrum of epithelial and mesenchymal properties may contribute to cancer progression, which typically lacks the coordinated and orderly induction of complete EMT. In the current review, however, the EMT theory of cancer cell motility forms the basis of discussion.

### Molecular Characteristics of EMT

At the molecular level, the characteristics of EMT include downregulation of epithelial markers, such as E-cadherin, desmoplakin, and cytokeratins, and upregulation of mesenchymal markers, such as *N*-cadherin, fibronectin, vimentin, and α-smooth muscle actin (α-SMA) (8, 14–17). Activation of transcription factors that include Snail, Twist, ZEB1 and ZEB2, and others, triggers EMT. These transcription factors repress the epithelial cell phenotype and promote mesenchymal characteristics of motility and BM and ECM degradation (14). For example, ZEB1 and ZEB2 directly bind to the promoter of target genes through conserved zinc finger proteins to downregulate expression of E-cadherin (epithelial marker) and induce expression of vimentin (mesenchymal marker) (15, 16). ZEB1 also enhanced the migration of PC-3 human prostate cancer cells through the extracellular barrier and increased metastatic colonization (16). Snail1 and Snail2, which belong to the Snail zinc finger family, are capable of repressing the expression of E-cadherin by directly binding to its promoter and activating the expression of proinvasive vimentin, fibronectin, and the matrix degradation enzymes termed matrix metalloproteinases (MMPs) (17, 18). Overexpression of Snail1 induced prostate cancer cells to degrade and break through BM barriers, thereby invading into the blood system, through directly activating membrane-anchored MMPs, MT1-MMP, or MT2-MMP (19). Snail1 also promoted angiogenesis, another essential process for tumor invasion and metastasis (20). Activated Twist is a member of the basic helix-loop-helix transcription factor family, and it plays a role in tumor invasion and metastasis *via* downregulating E-cadherin and upregulating N-cadherin (21). Twist also upregulates the expression of MMPs and downregulates the expression of tissue inhibitor of metalloproteinase, a naturally occurring specific inhibitor of MMPs (22).

### Circulating Tumor Cells

The movement of tumor cells to distant organ sites requires a risky journey with intravasion into the vasculature, immune attack, and extravasion to a new site for cancer development. Tumor cells that intravasate into the blood vessel lumens and disseminate to new sites of colony formation are termed circulating tumor cells (CTCs) (23). There is limited research on the association between CTCs, platelets, and macrophages; controls on transendothelial migration; and the molecular mechanisms active in these processes. The following section presents some information on the role of EMT in CTC production and motility. Perhaps the pre-eminent molecular pathway involves Notch signaling. The Notch pathway not only acts as a regulator of cell survival and cell proliferation but is also involved in cancer cell intravasation by stimulating transendothelial migration (23). How Notch signaling and EMT control the activity of CTCs is largely unknown. Nonetheless, the number of CTCs in blood is a significant prognostic factor for lung (23), colorectal (24, 25), breast (26), and prostate (27) cancers, and it is likely that CTCs feature in development of most carcinomas. They are found only rarely in normal healthy people and those with non-malignant tumors (28). EMT may help CTCs with cancer dissemination by overcoming detachment-induced cell apoptosis or “anoikis” and promoting survival in the circulation, but how EMT helps the CTCs is still not clear (29). In breast cancer, EMT may prevent CTCs from anoikis by enhancing cancer cell reattachment to leukocytes, platelets, and endothelium. EMT does this *via* formation of microtubule-based membrane protrusions on the CTCs, called microtentacles, which are formed on the surface of detached cells through expression of Twist1 and Snail1 (30, 31). In addition, neurotrophin receptor-interacting melanoma antigen (NRAGE) interacts with ankyrin-G, a part of the E-cadherin complex, rendering cancer cells sensitive to apoptosis. During oncogenic EMT, loss of E-cadherin downregulates ankyrin-G, enhancing NRAGE translocation to the nucleus, where the NRAGE-TBX2 complex can inhibit p14ARF gene expression to protect cancer cells against anoikis (32).

Some of the surviving CTCs that move through the vascular lumen and lodge on the vascular endothelium may break through the endothelial and pericyte layers and then enter the tissue parenchyma at the secondary organ site. During this process, the adherent cancer cells must communicate with endothelial cells to open their cell junctions, thus allowing the passage of the cancer cells to extravasate across the endothelium to the connective tissue space of the host organ (33). Cancer cells can migrate through the endothelial layer of blood vessels in two basic ways: paracellular migration, where cancer cells cross the endothelial layer by disrupting the cell junctions in the endothelial layer; and transcellular migration by which cancer cells cross the endothelial barriers by traversing through the endothelial cell body (10, 34). An extravasation assay in zebrafish was used to model the roles of EMT in the process of metastasis. Zebrafish are transparent and allow real-time imaging of cell movement in the live animal. The results demonstrated that Twist1, a central protein in EMT, affects intravascular migration of arrested cancer cells, remodels the vasculature, and promotes cancer cell exit from the blood circulation through a β1 integrin-independent mechanism (35). On extravasation into a foreign microenvironment, tumor cells follow one of three alternative courses: cell death, dormancy, or senescence.

**Figure 1:**
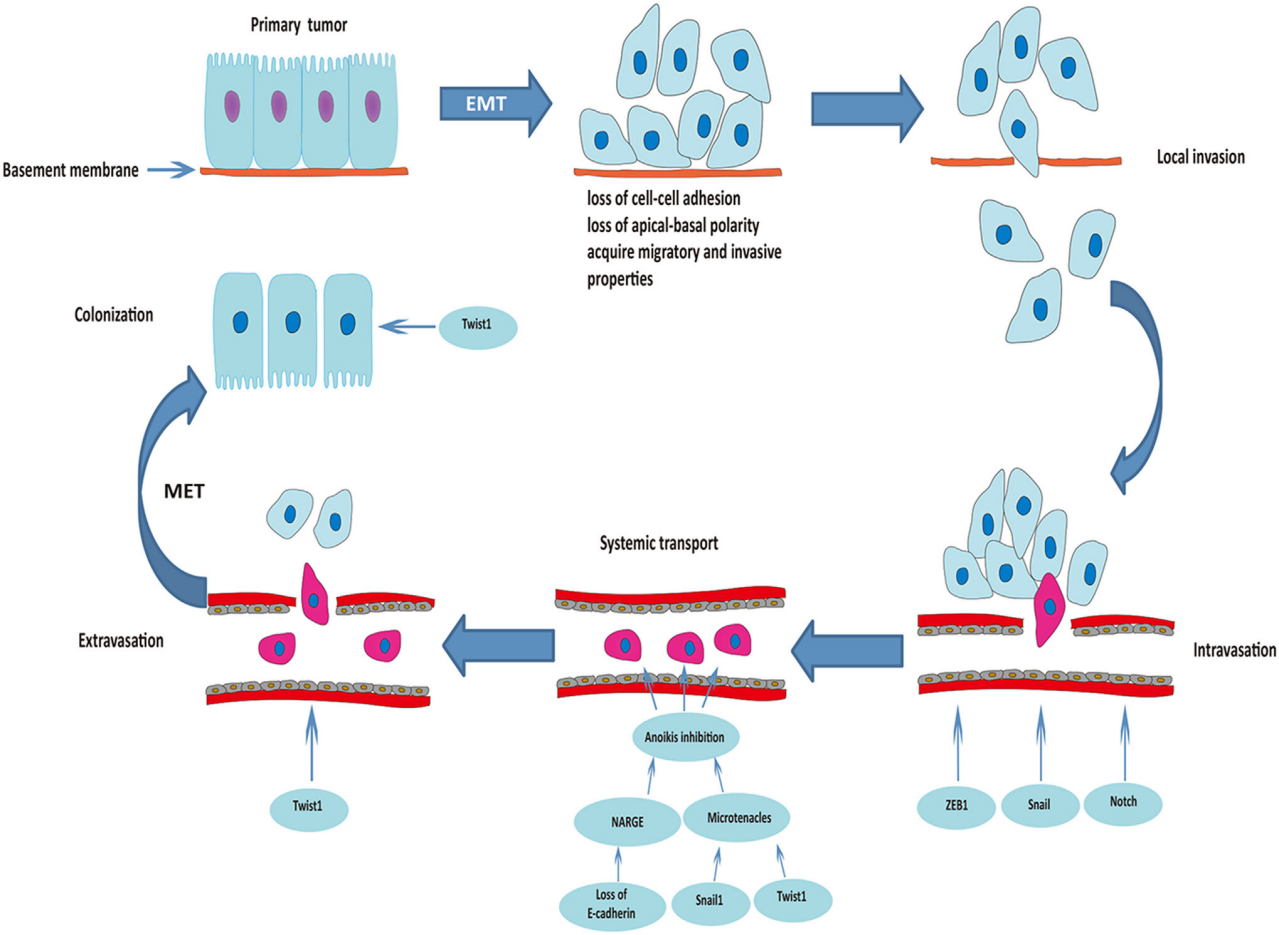
Roles of epithelial–mesenchymal transition (EMT) in the process of cancer metastasis. Triggered by the activation of transcriptional factors, tumor cells of epithelial origin that undergo EMT are transformed into a mesenchymal phenotype, lose cell-to-cell adhesion and apical-basal polarity, and acquire migratory and invasive properties. After degradation of the underlying basement membrane and extracellular matrix, tumor cells invade the neighboring tissue parenchyma (local invasion). The mesenchymal–phenotype tumor cells then invade into the blood or lymphatic vessels (intravasation). ZEB1, Snail, and Notch are involved in this process. Circulating tumor cells (within vessel lumens) overcome the harsh conditions in the blood stream and attach to the vessel wall at a distant site to prepare for escape from the blood circulation. Transcriptional factors Snail1 and Twist1 play an important role in preventing anoikis and maintaining survival of circulating tumor cells. Some of the surviving circulating tumor cells break through the vascular wall and enter the tissue parenchyma at the secondary organ site (extravasation). Finally, some of the EMT-derived mesenchymal cells with cancer stem cell-like properties undergo mesenchymal–epithelial transition (MET) to initiate proliferation of the metastatic clone.

Occasionally, EMT-derived mesenchymal cells with CSC-like properties (less than 1% of disseminated cancer cells) undergo MET to be able to initiate cancer cell proliferation and differentiation during colonization (34, 36). Downregulation of Twist1 at the metastatic site was essential for increased proliferation and reversal of EMT-induced growth arrest, which clearly proves the indispensable role of MET for colonization and macrometastasis (37). Figure 1 summarizes the roles of EMT in the process of metastasis.

## Role of TAMs in Tumor Metastasis

**Figure 2:**
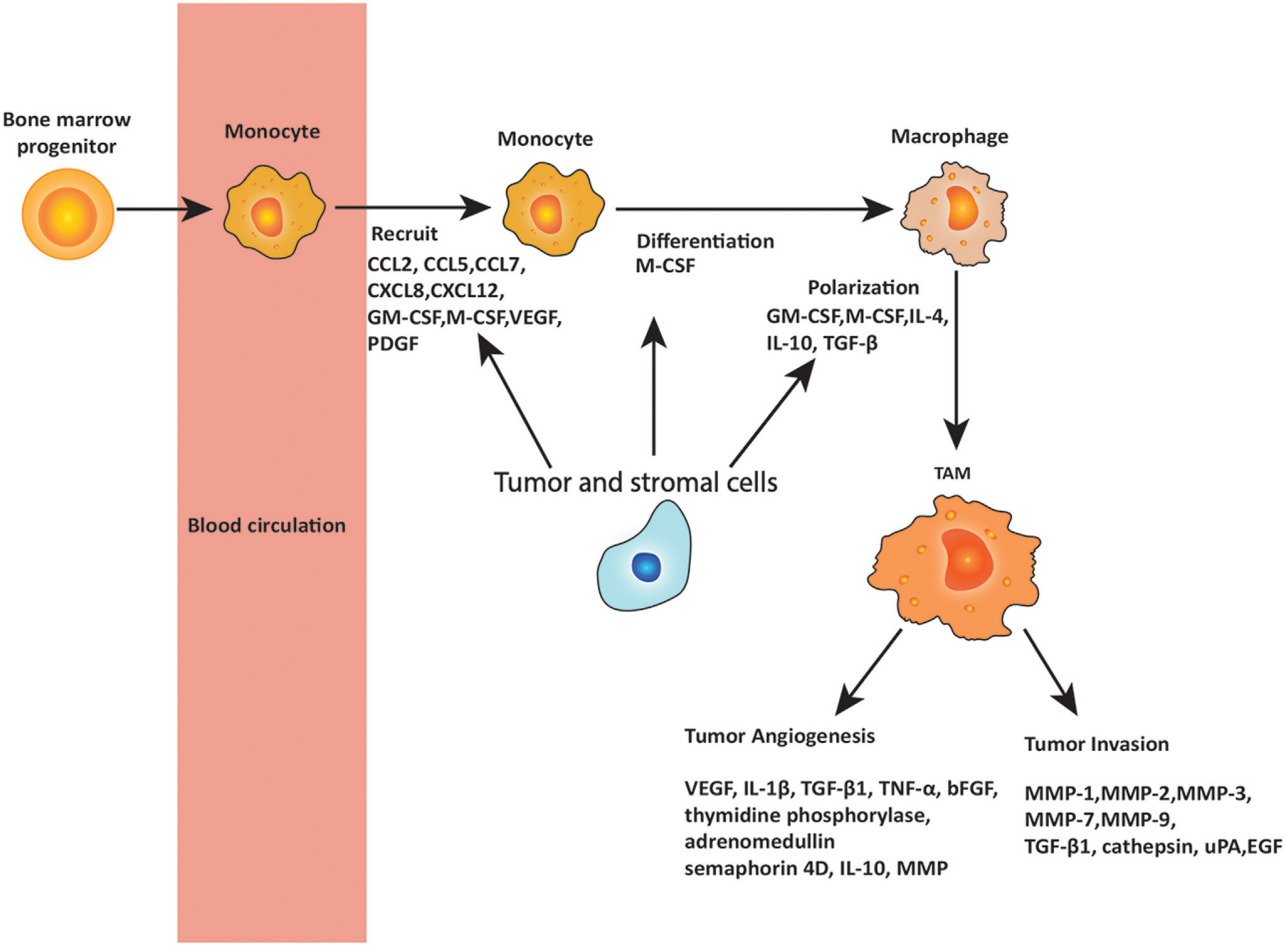
Roles of tumor-associated macrophages (TAMs) in metastasis. Circulating monocytes that are derived from bone marrow progenitors are recruited to the tumor site by chemokines and cytokines secreted by tumor and stromal cells. These chemokines and cytokines include C-C chemokine motif ligand 2 (CCL2), CCL5, CCL7, C-X-C chemokine motif ligand 8 (CXCL8), and CXCL12, as well as granulocyte–macrophage colony-stimulating factor (GM-CSF), macrophage colony-stimulating factor (M-CSF), vascular endothelial growth factor (VEGF), and platelet-derived growth factor (PDGF), of which CCL2 is the most important. In the tumor microenvironment, monocytes are differentiated and polarized to TAMs by the cytokines produced by tumor and stromal cells, including M-CSF, GM-CSF, interleukin 4 (IL-4), IL-10, and transforming growth factor-β (TGF-β). TAMs play important roles in promoting tumor angiogenesis and invasion through releasing various cytokines including VEGF, IL-1β, TGF-β1, matrix metalloproteinases (MMPs), tumor necrosis factor (TNF)-α, basic fibroblast growth factor (bFGF), IL-10, urokinase plasminogen activator (uPA), epidermal growth factor (EGF), and others as noted in the figure.

The role of macrophages in cancer is controversial, and many aspects remain unresolved. Macrophage surveillance is essential for preventing the cancer growth, and there is evidence that activated macrophages can identify and kill transformed cells. However, there is also evidence that macrophage depletion has little effect on the host’s susceptibility to cancer (38) and in some cases may even be beneficial to the host. Different subpopulations of macrophages may be responsible for distinct tumor-promoting activities and tumor relapse on different types of therapies. Here, the proposal that TAMs have contrasting roles in cancer depending on their phenotype is discussed. Figure 2 summarizes the role of TAMs in cancer development.

### Macrophage Differentiation and Polarization

Macrophages are differentiated immune cells of the myeloid lineage, found in all tissues (39). Most tissue-resident macrophages originate from the embryonic yolk sac and demonstrate tissue-specific functional properties important for the tissue differentiation and homeostasis. To carry out their tissue-specific functions, macrophages respond to local signals released in their niche. They also act as sentinel cells that constantly monitor their tissues for potential threats, such as infection, injury, and tumors, and they respond rapidly by initiating the inflammatory cascade. During inflammation, macrophages also differentiate from circulating monocytes that recruit to the site of inflammation and extravasate into the tissue (40). Importantly, macrophages also play a fundamental role in promoting the resolution of inflammation by clearing apoptotic cells/debris and supporting wound healing and tissue repair programs (41–43). When resident and recruited macrophages activate in response to potentially harmful agents, they can acquire different polarization states. Historically, according to their activation state, macrophages divide into classically activated M1 and alternatively activated M2 macrophages (44, 45). This classification reflects the Th1/Th2 paradigm of T helper cell activation. M1 macrophages, typically, activate with IFN-γ, lipopolysaccharides, viral products, or granulocyte-macrophage colony-stimulating factor (GM-CSF). In contrast, M2 macrophages, typically, activate with IL-4, IL-10, glucocorticoids, or macrophage colony-stimulating factor (M-CSF) (46, 47). M2-like macrophages have been often further divided into M2a (IL-4 induced), M2b (IgG-induced), and M2c (IL-10 and glucocorticoid-induced). More recently a consensus was reached to, instead, define macrophage phenotypes based on the activator used (48, 49). It is important to note that these polarization states are just extremes of a continuum *in vivo*. Macrophages display a high degree of plasticity, and the different activation states can often coexist or change during disease progression (50). Moreover, according to Mills, who originally developed the M1/M2 paradigm, what really matters is the function that macrophages play within a specific *in vivo* context: M1/kill and M2/repair (51). Therefore, M1 macrophages were defined originally *in vivo* by the production of the functional molecule nitric oxide that inhibits proliferation. M1 responses are linked with IL-12 and IL-8/CCL (C-C chemokine motif ligand) production, and cell surface expression of CD80 or 86 that attract killer cells like neutrophils and/or stimulate Th1 responses such as cytotoxic T lymphocytes (52–54). M2 macrophages were defined originally *in vivo* by the production of ornithine that promotes proliferation. Their responses are associated with transforming growth factor (TGF)-β and other growth factor production, for example, vascular endothelial growth factor (VEGF) or epidermal growth factor (EGF), cell surface expression of CD163 or 206, and the propensity to stimulate Th2 responses such as antibody production and antibody-dependent cell-mediated cytotoxicity. Many macrophage populations express additional cytokines and growth factors, including tumor necrosis factor (TNF)-α, IL-6, IL-1, IL-10, NADPH oxidases, and MMPs. Changes in the relative ratios between pro-inflammatory and immunosuppressive/remodeling factors characterize a more M1-like or M2-like polarization state (55).

### Tumor-Associated Macrophages

Tumor-associated macrophages represent the major type of immune cells that infiltrate the tumor microenvironment (56–58). Over the last decades, many studies have shown a significant link between TAM number/density and a poor prognosis in most tumor types, illustrating the clinical significance of macrophages in tumor progression (58). For example, there is a significant association between higher risk of distant metastasis and poor overall survival with increased density of TAMs in renal cell (59), breast (60, 61), bladder (62), and hepatocellular (63) cancers, and lymphoma (64, 65). It is now clear that dynamic changes in the phenotypes of macrophages occur during the different steps of tumor progression, including invasion, metastasis, seeding, and growth (66). Different subpopulations of TAMs are responsible for distinct tumor-promoting activities and tumor relapse on different types of therapies (for example, irradiation, antiangiogenic therapies, some forms of chemotherapy) (67–70). Currently, it is not known if TAMs derive from resident or recruited macrophages. Small tumors probably interact with the surrounding resident macrophages and, only later as the tumor mass grows and becomes vascularized, are circulating monocytes recruited and differentiate into TAMs. Circulating monocytes usually derive from bone marrow progenitors; however, under abnormal circumstances, such as during tumor development, extramedullary hematopoiesis can also take place in the spleen (71). Peripheral blood monocytes consist of two main populations: inflammatory or classical monocytes (Ly6C^high^CX3CR1^low^ CCR2^high^ in mouse and CD14^++^CD16^neg^ in humans) and patrolling or non-classical monocytes (Ly6C^low^ CX3CR1^high^ CCR2^low^ and CD14^+^CD16^++^). Many chemokines and cytokines secreted by tumor and stromal cells recruit peripheral blood monocytes to the tumor site. These include CCL2, CCL5, CCL7, CXCL8, and CXCL12, as well as GM-CSF, M-CSF, VEGF, and platelet-derived growth factor (71, 72). Among these chemokines, CCL2 is likely the most important in recruitment of TAMs. However, the monocyte subset that recruits to the primary tumor and the involvement of the CCL2-CCR2 chemokine axis appears to be model and tumor type dependent (73).

Within the tumor microenvironment, monocytes are induced to differentiate and polarize into protumoral macrophages through complex signaling networks induced by multiple soluble mediators, such as M-CSF, GM-CSF, and immunosuppressive cytokines such as IL-4, IL-10, and TGF-β (74). TAMs respond by modulating cancer cell proliferation, immune regulation, ECM remodeling, tumor cell invasion and metastasis, lymphangiogenesis and vascular angiogenesis by secreting IL-10, VEGF, prokineticin (Bv8), prostaglandin E2, and MMP-9 (75–77). As the tumor progresses, TAMs are more and more polarized toward an M2-like phenotype and express a typical M2 signature including high levels of MR (CD206), scavenger receptor-A, arginase-1, and low MHC-class II antigens. The roles played by TAMs in promoting tumor angiogenesis, tumor invasion, as well as intravasation, extravasation, and seeding of metastatic tumor cells are relevant to this review.

### TAMs and Tumor Angiogenesis

There is considerable controversy about whether TAMs promote tumor progression and metastasis *via* their stimulation of tumor angiogenesis (78). An angiogenic switch is crucial for tumor cell survival, proliferation, invasiveness, and metastasis. High levels of TAM infiltration in the primary tumor are usually associated with angiogenesis *via* upregulation and release of several pro-angiogenic factors that, typically, promote new vessel growth. For example, VEGF-A is an important pro-angiogenic cytokine secreted by TAMs. Under hypoxic conditions, hypoxia-inducible factors (HIF-1α and HIF-2α) increase the production of VEGF-A from TAMs and, thus, promote TAM-induced angiogenesis and metastasis (79). Under normoxic conditions, IL-1β and TGF-β1 released by TAMs promote the expression of VEGF-A in mouse macrophages *via* HIF-1α/β- and Smad3/4-dependent signaling pathways (80–83). Other pro-angiogenic factors secreted by TAMs, including TNF-α, basic fibroblast growth factor, thymidine phosphorylase, adrenomedullin, and semaphorin 4D, are also involved in angiogenesis and metastasis (84, 85). A specific subpopulation of pro-angiogenic TAMs was identified, characterized by the expression of the angiopoietin receptor Tie2, and endowed with pro-angiogenic and immunosuppressive activities (86, 87). Tie2-expressing monocytes/macrophages express higher levels of CD163 and CD206, indicating that Tie2-expressing myeloid cells show an M2-like phenotype. They also express VEGF, IL-10, and MMP-9. Tie2-expressing monocytes/macrophages recruit to tumors and are required for tumor growth. This makes them excellent candidates for the development of new tumor targeted therapies. Indeed, targeting the angiopoietin 2 (ANG2)/Tie2 pathway, with an anti-ANG2 antibody, inhibits tumor growth and metastasis by disabling the pro-angiogenic activity of Tie2-expressing monocytes/macrophages and impeding the emergence of evasive resistance to antiangiogenic therapy (88).

### TAMs and Tumor Invasion

The tumor microenvironment is important for tumor progression. Among the components of the microenvironment, TAMs are thought to be the major inflammatory component of the tumor support stroma. However, there remain inconsistent data on whether TAMs promote tumor invasion. Here, we present some evidence for a positive link between TAMs and tumor invasion.

Matrix metalloproteinases are a family of zinc-dependent proteases that function to degrade matrix, which includes collagenase (MMP-1), gelatinase A (MMP-2), stromelysin (MMP-3), matrilysin (MMP-7), gelatinase B (MMP-9). MMPs are involved in tumor invasion and metastasis by degrading the BM, activating growth factors, and promoting angiogenesis (89, 90). Macrophage-derived TGF-β1 can promote MMP-9 expression in glioma stem-like cells, thereby enhancing the invasiveness of tumor cells (81). Moreover, specific ablation of MMP-9-positive TAMs with zoledronic acid resulted in reduced angiogenesis (91, 92). Macrophages are also effective producers of additional proteases, including cathepsins and serine proteases such as urokinase-type plasminogen activator. In many tumors, these proteases function to enhance tumor progression and metastasis by proteolytic destruction of the matrix to allow tumor cells to escape from the confines of the BM and to migrate through the dense stoma (93–95).

An important EGFR/CSF-1R paracrine loop exists between macrophages and tumor cells in which CSF-1, produced by carcinoma cells and bound by macrophages, promotes the proliferation, differentiation, and polarization of macrophages toward an M2-like phenotype (96). CSF-1 also stimulates macrophages to release EGF, which promotes tumor cell proliferation and migration. EGF also stimulates the secretion of CSF-1 by tumor cells, thereby forming a positive feedback loop between tumor cells and macrophages (97, 98). Recently, intravital microscopy was used to investigate tumor cell migration toward blood vessels in mouse models of breast cancer (99). By using live cell imaging, perivascular Tie2-expressing macrophages promoted the transient opening of tumor blood vessels in a VEGF-dependent manner. Diffusion of plasma proteins from leaky vessels attracts cancer cells that can easily enter systemic circulation at the open gates (99). Previous work from this group had identified perivascular structures called “tumor microenvironment of metastasis” in which a macrophage, a specialized cancer cell, and a blood vessel establish tripartite contacts. Importantly, “tumor microenvironment of metastasis” cell density predicts distant metastatic recurrence in breast cancer patients independently of other clinical prognostic indicators (100). TAMs are also major contributors in the formation of the metastases at secondary sites of primary tumor, where they prepare a suitable microenvironment for successful colonization (101).

## Crosstalk Between TAMs and EMT in Tumor Metastasis

The interactions of cancer cells with various stromal cells in the tumor microenvironment, as well as the accumulation of intrinsic changes in cancer cells, drive progression and metastasis. The tumor microenvironment comprises endothelial cells, pericytes, fibroblasts, ECM, and various bone marrow-derived cells such as macrophages, neutrophils, and mast cells. As a major component of the tumor microenvironment, TAMs act in cancer migration, invasion, and metastasis through processes such as EMT by producing or activating various factors including nuclear factor-κB (NF-κB) and important inflammatory cytokines and growth factors, such as IL-6, TGF-β1, TNF-α, and IL-8 (102). Information on these co-activating factors is now provided.

### Factors That Contribute to Tumor Metastasis

#### Nuclear Factor-κB

Nuclear factor-κB consists of a family of transcription factors that play pivotal roles in both TAMs and tumor cells to promote tumor initiation and growth (103, 104). NF-κB activation is also an important event in the acquisition of metastatic potential in cancer cells, and its activation can result from the crosstalk between TAMs and cancer cells. For example, TAMs prime NF-κB activation in both stromal and cancer cells by secreting TNF-α, which in turn upregulates Snail expression (105). In the process of TNF-α-induced EMT, NF-κB regulates phosphorylation and degradation of Snail. Knockdown of Snail by shRNA was able to inhibit migration and invasion of breast cancer cells induced by TNF-α *in vitro* as well as inflammation factor-mediated metastasis *in vivo* (106). TAMs also promoted tumor invasion and migration ability and transformation of oral squamous cell carcinoma cells from an epithelial-to-mesenchymal phenotype through activation of the Gas6/Axl-NF-κB signaling pathway (105).

#### Interleukin-6

Interleukin-6 is an inflammatory cytokine upregulated in most common human cancers, with increased levels of IL-6 in serum indicating poor prognosis in most cancers (107). IL-6 decreased the expression of E-cadherin in estrogen receptor α-positive human breast cancer cells, with constitutively ectopic IL-6-expressing MCF-7 breast cancer cells (MCF-7^IL-6^) exhibiting an EMT phenotype characterized by decreased expression of E-cadherin and upregulation of vimentin, N-cadherin, Snail, and Twist (108). *In vitro* co-culture of human lung adenocarcinoma A549 or H1299 cells with THP-1-derived macrophages upregulated IL-6 and increased the invasion ability of A549 and H1299 cells through EMT by repressing the expression of E-cadherin and promoting vimentin. In addition, the presence of additional anti-IL-6 antibody was able to neutralize the enhanced invasiveness (109).

#### Transforming Growth Factor-β1

Transforming growth factor-β1 is an important inflammatory cytokine that is involved in inducing EMT, facilitating tumor cell evasion of immune surveillance, and promoting cancer cell dissemination and metastasis (110). In the tumor microenvironment, TGF-β1 is produced by infiltrating immune cells such as TAMs, myeloid-derived suppressor cells and regulatory T cells, and cells that are major promoters of EMT (7). TGF-β1 derived from TAMs induced and promoted EMT in various cancer cells (111, 112). TGF-β1 also promoted cancer stem cells-like properties in hepatocellular carcinoma Hepa1-6 cells, which underwent EMT and acquired higher invasive ability (113). Once secreted, TGF-β1 can function in an autocrine manner to sustain the mesenchymal and stem cell traits of cancer cells that are undergoing EMT (114).

#### Tumor Necrosis Factor-α

Macrophages are major producers of TNF-α and are highly responsive to TNF-α. High levels of TNF-α can lead to anticancer effects through activating T cell-mediated immunity, whereas low-level, chronic TNF-α produced by cancer cells and stromal cells may promote tumor growth and metastasis (115). TNF-α can induce EMT and promote tumorigenicity of renal cell carcinoma and increase invasion and migration activities by inhibiting E-cadherin, upregulating vimentin expression and activating MMP-9. The PI3K/Akt/GSK-3β signaling pathway plays an important role in the TNF-α-induced EMT processes of in renal cell carcinomas (116). This knowledge may be a key to targeting TNF-α receptors to promote antitumor immunity. The design of therapeutic antibodies, which engage and activate TNF-α receptors for cancer therapeutics but avoid serious immune-related adverse events, is a current challenge (117).

#### Interleukin-8

Interleukin-8, also known as CXCL8, plays a vital role in cancer progression by initiating angiogenesis; recruiting monocytes to the tumor site; and enhancing the proliferation, survival, and metastasis of cancer cells (118, 119). The mechanism of IL-8 involvement in EMT is that Snail activates the expression of IL-8 by binding to its E3/E4 E-box located in the promoter of IL-8 (120). There is also a link between IL-8 secreted by TAMs and tumor EMT. For example, TAMs induce EMT of hepatocellular carcinoma cells through the IL-8 activated JAK2/STAT3/Snail signaling pathway (120, 121).

#### Other Contributors

Tumor-associated macrophages also promote tumor cell invasion and metastasis *via* the interactions with cancer stem cells. For example, during EMT, increased expression of CD90 and EphA4 control the cell-to-cell interactions between TAMs and cancer stem cells through directly binding with their respective receptors on the surface of these cells (122). For example, Lewis lung carcinoma cells potently activated macrophages leading to production of IL-6 and TNF-α through activation of the TLR family members TLR2 and TLR6 (123). Endothelial activation markers, including vascular cell adhesion molecule-1 (VCAM-1) and vascular adhesion protein-1 (VAP-1), are induced near metastatic tumor cells after their attachment to the vascular bed. Embolus formation of tumor cells enhances VCAM-1 induction. Blocking endothelial activation, with either an anti-VCAM-1 blocking antibody or a VAP-1 inhibitor, resulted in decreased recruitment of macrophages and reduced metastatic cell survival (124).

## Breaking Crosstalk Between TAMs and EMT for the Treatment of Tumor Metastasis

Are insights gleaned from basic laboratory research useful to the diagnosis and treatment of clinical metastatic disease? The interaction and crosstalk between TAMs and EMT undoubtedly promotes tumor progression, invasiveness, and metastasis, at least in some cancers. As mentioned previously, secretion of various cytokines and chemokines by TAMs orchestrates the crosstalk and promotes adjacent epithelial tumor cells to undergo EMT through a paracrine manner. In turn, cytokines produced by tumor cells also promote the differentiation process in TAMs, thereby forming a positive feedback loop between TAMs and EMT in metastasis (125). Thus, breaking the pathways of crosstalk between TAMs and EMT is a reasonable option for the treatment of tumor metastasis.

In terms of targeting TAMs; several strategies have been promising: blocking recruitment of TAMs; depleting numbers of TAMs by promoting their removal; reversal of immune suppression by switching the M2 to M1 phenotype; and inhibiting TAM-induced tumor angiogenesis (126). For example, blockade of TAM recruitment by the genetic deletion of CSF-1 delayed tumor growth, angiogenesis, and reduced metastasis in several models of cancer (127). Inhibition of CSF-1 or its receptor with antibody, small molecule inhibitors, or antisense RNA reduced the recruitment of macrophages, thereby inhibiting the proliferation of cancer cells and metastasis (128–130). Specific depletion of macrophages in the tumor microenvironment by clodronate-encapsulated liposomes or zoledronic acid significantly inhibited tumor proliferation, angiogenesis, and distant metastasis in liver, lung, and xenograft models of colon carcinoma (131–134). In a breast cancer model, the combination of CpG with an anti-IL-10 receptor antibody switched TAMs from the M2 to the M1 phenotype and triggered an innate immune response of debulking of tumors (135). Trabectedin, a chemotherapeutic agent approved in Europe for the treatment of sarcomas and ovarian cancer, was selectively toxic for TAMs, resulting in reduction of angiogenesis in mouse tumor models (136).

Since EMT is a crucial step in the process of cancer metastasis, targeting transcriptional factors of EMT or EMT-related pathways by miRNA has proven to be an effective strategy for the treatment of tumor metastasis. For example, members of the miR-200 family have emerged as pivotal repressors of EMT and cancer metastasis through ZEB-dependent and ZEB-independent mechanisms (137, 138). Delivery of miR-200 members to different cancer models, including lung, ovarian, renal, and basal-like breast cancers, led to significant reduction in primary tumor burden and distant metastasis (139). Cortez et al. have reported that transiently transfected lung cancer cell line A549 with miR-200c resulted in significantly more sensitivity to the cytotoxic effects of radiation. Systemic delivery of miR-200c enhanced the effects of radiotherapy in a xenograft model of lung cancer (140). The miR-34 family also plays an important role in reducing the viability of cancer stem cells and inhibiting metastasis through downregulating the expression of Snail in a manner of double-negative feedback loop (141, 142). Chen et al. have reported that systemic delivery of miR-34a into experimental lung metastasis models of B16F10 melanoma resulted in the downregulation of the survivin gene and reduction of tumor load in the lung (143).

Given the increasing evidence supporting active crosstalk between TAMs and EMT in tumor metastasis, targeting the signaling pathways in this process is another option for the treatment of invasive cancer. As mentioned above, the signaling pathway induced by TGF-β plays an important role in this crosstalk. Therefore, TGF-β-induced signaling cascades are potential therapeutic targets. For example, CX-4945, a potent and selective inhibitor of protein kinase CK2, inhibited the EMT-mediated migration and invasion of A549 human lung cancer cells by blocking the TGF-β1 signaling pathway (144). Ginsenoside 20 (R)-Rg3, an active component of ginseng, suppressed A549 cell migration and invasion through inhibiting the EMT process induced by TGF-β1 (145). Sorafenib, a tyrosine kinase inhibitor used for the treatment of primary kidney cancer, advanced primary liver cancer, and radioactive iodine-resistant advanced thyroid carcinoma inhibited TGF-β released in TAMs; reduced TGF-β-induced cancer cell proliferation, metastasis, and EMT process *in vitro;* and partially blocked macrophage activation *in vivo* (146).

Because NF-κB is another crucial cytokine involved in the crosstalk between TAMs and cancer cell EMT, targeting the NF-κB pathway has become one of the intensely studied strategies for the treatment of TAM-related tumor metastasis. Zoledronic acid is a third-generation bisphosphonate for the treatment of bone metastasis in breast cancer patients and for osteoporosis. Zoledronic acid reversed EMT in triple-negative breast cancer cells by inactivation of NF-κB signaling (147). A somatostatin derivative (smsDX) can block the paracrine loop between TAMs and prostate cancer cells and reduce the risk of migration and invasion through inhibiting activation of NF-κB (148).

## Conclusion and Outlook

A major complication of cancer progression is the metastatic spread of cancer cells from the primary tumor. Similarities between the process of EMT in embryogenesis and wound healing and that seen in spread of epithelial-derived cancers are now becoming clear. There is increasing evidence that the tumor metastatic cascade relies on a complicated interaction between EMT-modified cancer cells and TAMs. Cytokines released by tumor cells promote the recruit of macrophages to the tumor site and differentiate them into TAMs that then become active in the tumor microenvironment. Reciprocally, as a major component of solid tumors, TAMs promote cancer cell invasion and metastasis by secreting various cytokines, for example, TGF-β, NF-κB, VEGF, and CCL18. Thus, a positive feedback circuit forms between TAMs and EMT-modified cancer cells in the tumor microenvironment (125). Despite some residual gaps in knowledge regarding links between EMT, TAMs, and cancer invasiveness, it appears that targeting transcriptional factors and the signaling pathways between TAMs and EMT can break the cycle of crosstalk to combat metastasis. Although most evidence detailed here was from experimental models, some results involving patient-derived cancers support the preclinical experimental data. With further elucidation about the mechanism of tumor metastasis, it may soon be possible to translate these fundamental research discoveries successfully into clinic practice.

## Author Contributions

WS, RM, TY, and GG contributed equally to deciding on topics of interest in this manuscript. WS wrote the manuscript, and all authors contributed equally to its editing.

## Conflict of Interest Statement

The authors declare that the research was conducted in the absence of any commercial or financial relationships that could be construed as a potential conflict of interest.
